# High variability of COVID-19 case fatality rate in Germany

**DOI:** 10.1186/s12889-023-15112-0

**Published:** 2023-03-02

**Authors:** Matthias Wjst, Clemens Wendtner

**Affiliations:** 1 Institut für Lungenbiologie (ILBD), Helmholtz Zentrum München, Deutsches Forschungszentrum für Gesundheit und Umwelt (GmbH), Ingolstädter Landstr. 1, 85764 München-Neuherberg, Germany; 2 Institut für KI und Informatik in der Medizin, Lehrstuhl für Medizinische Informatik, Klinikum rechts der Isar, Grillparzerstr. 18, 81675 München, Germany; 3 München Klinik, Klinikum München Schwabing, Kölner Platz 1, 80804 München, Germany

**Keywords:** COVID-19, Case fatality rate, High flow nasal cannula, Mechanical Ventilation, Munich, Hamburg, Germany

## Abstract

**Background:**

During the first wave of the COVID-19 pandemic a high case fatality rate (CFR) was noticed worldwide including also Germany where the first European cases have been observed. The WHO recommended immediate intubation for patients with dyspnoea which has since been revised after reviewing the initial clinical outcome. The objective of this study is to analyze CFR and assess if there is an advantage of a more conservative management of COVID-19 induced hypoxemia.

**Methods:**

PCR confirmed COVID-19 infections and death counts were obtained for all German districts from 27 Jan 2020 until 15 Feb 2021 using official reports of Robert Koch Institute Berlin, Germany. A moving average CFRt was constructed by dividing disease related deaths two weeks after a given index day by the number of infections two weeks prior to that date. In addition to a local comparison also mortality outcomes in other German speaking countries were compared.

**Results:**

The mean CFR is estimated to be 2.92% based on 71.965 fatalities and 2.465.407 cases. There was a large regional scattering of CFRs across the German districts. Differences of the mortality pattern were observed also at state level and preserved across different sex and age groups while being largely independent of case numbers. Although Munich city had higher infection rates, more patients died during the first wave in Hamburg (OR 1.6, 95% CI 1.3–1.9) which was mirrored also by higher death risk at Hamburg intensive care units (OR 2.0, 95% CI 1.3–3.1). While the majority of Munich hospitals favoured a conservative management of hypoxemia including high flow nasal cannula (HFNC), Hamburg hospitals followed a more aggressive scheme of early mechanical ventilation (MV). Austria and Switzerland experienced higher CFRs than Germany during the first wave but after changing their treatment guidelines, both countries experienced lower CFRs during the second wave.

**Conclusion:**

Using retrospective public health data, different case fatality rates have been observed across Germany. A more conservative management of COVID-19 induced Adult Respiratory Distress Syndrome (ARDS) is justified also by epidemiological data.

**Supplementary Information:**

The online version contains supplementary material available at 10.1186/s12889-023-15112-0.

## Background

The COVID-19 pandemic wave started in Europe by the end of January 2020 [1]. While the initial cluster could be successfully contained [2, 3] the infection resurfaced only a month later in Bavaria (Germany), in the canton of Ticino (Switzerland) as well as in Tirol (Austria). An exceptional high case fatality rate (CFR) was observed at hotspots like Wuhan (China), Bergamo (Italy), and New York (US) [4]. An outbreak at the Diamond Princess cruise ship showed an age adjusted case fatality ratio as high as 2.6% (95% confidence interval (CI): 0.9–6.7) [5].

A high COVID-19 mortality has also been described in Germany at population level [6]. Another study, more from a clinical perspective examined 10.021 cases admitted to German hospitals. This study reported an in-hospital mortality of 22% and a huge variation depending on type of ventilation [7]. Both WHO and German guidelines recommended immediate intubation and mechanical ventilation (MV) as primary treatment of severe cases, although this recommendation was based on rather weak evidence [8]. As more than half of all patients died under MV, clinicians already in April 2020 raised doubts about the existing treatment guidelines.

Disease management has since undergone major changes. The discussion how COVID-19 related ARDS should be treated—either more aggressively by early intubation and MV or more conservatively by applying non-invasive ventilation (NIV) and high flow nasal cannula (HFNC) [9] —has since been under substantial debate. The objective of this study therefore is to further analyze CFR and possibly answer the question if there is an advantage of a more conservative management of COVID-19 induced hypoxemia.

## Methods

The analysis relies on a COVID-19 surveillance project [1] that started after the identification of the first European case in Munich, Germany on Jan 27, 2020 and includes only laboratory confirmed disease reports. The end date for this analysis is 15 Feb 2021 just before the upsurge of the new variant B.1.1.7. and after the emergency use authorisation of the first vaccines in Germany by December 2020. The first and second waves were separated visually by 15 Aug 2020.

Regional GPS data were obtained from public.opendatasoft.com (28 March 2020), district data from www-genesis.destatis.de/genesis/online (29 March 2020) and hospital beds from www.landatlas.de/download/E/Krankenhausbetten.xlsx (2 Jan 2021). Worldwide COVID-19 case and death counts were downloaded from github.com/owid/covid-19-data/tree/master/public/data (15 Feb 2021).

Daily German COVID-19 case and death counts have been publicly released by Robert Koch Institute in Berlin opendata.arcgis.com/datasets/917fc37a709542548cc3be077a786c17_0.csv using the data freeze 6 March 2021. Two district codes have been corrected due to political reorganization while 12 areas of Berlin have been combined into one large area resulting in 402 districts under analysis. All database entries with error codes were deleted. Clinical data were obtained from the public DIVI register ("Deutsche interdisziplinäre Vereinigung für Intensiv- und Notfallmedizin") that had been started by end of March 2020. Since the database was not fully functional before May 2020, only second wave data could be included here from www.datawrapper.de/_/wwQvR starting on Oct 31, 2020. Patient numbers of München hospitals were obtained from the local clinical management system (München Klinik Schwabing, TU München and LMU Koordination Pandemiemanagement) while data of Hamburg hospitals were retrieved from the literature [10] including the time interval 27 Jan 27 2020 until 3 June 2020. As PCR reagents had been temporarily exhausted, underreporting is being expected at some time points during the observation period.

The CFR is usually defined as the proportion of cases of a specified condition that are fatal within a specified time. This definition may lead to a paradox that more persons die of a disease than develop it during a given time period [11]. CFR therefore can be considered only final after the end of a pandemic although there maybe a need to calculate a CFRt even earlier as an evaluation parameter of interventions. Analogously to SIR infection models [12], CFRt has been constructed here by using a moving average where cases during the two weeks before the index day are summed and used as reference to all fatalities during the two weeks following the index day [13, 14]. Nevertheless, whenever infection rates fall too low, CFR values become unreliable high.

As the current analysis is neither preregistered nor does it include any sample size calculation, no P-values are reported in accordance to recent recommendations [15]. Instead graphical displays are used to describe the temporal trends [16–18] and whenever possible, 95% confidence intervals, have been included. R Version 3.6.3. was used along with Rstudio 1.3.1093 along with packages ggplot2, rgdal, ggmap, openxlsx, ggrepel, gmodels, MASS, dplyr, grid, gridExtra, rgeos, sp, sf, openxlsx, rjson, tidyverse, stringr, magrittr and epitools. As mainly public data have been included, no ethical board has been involved. Nevertheless all methods were performed in accordance with the relevant ethical guidelines and local regulations.

## Results

**Fig. 1:**
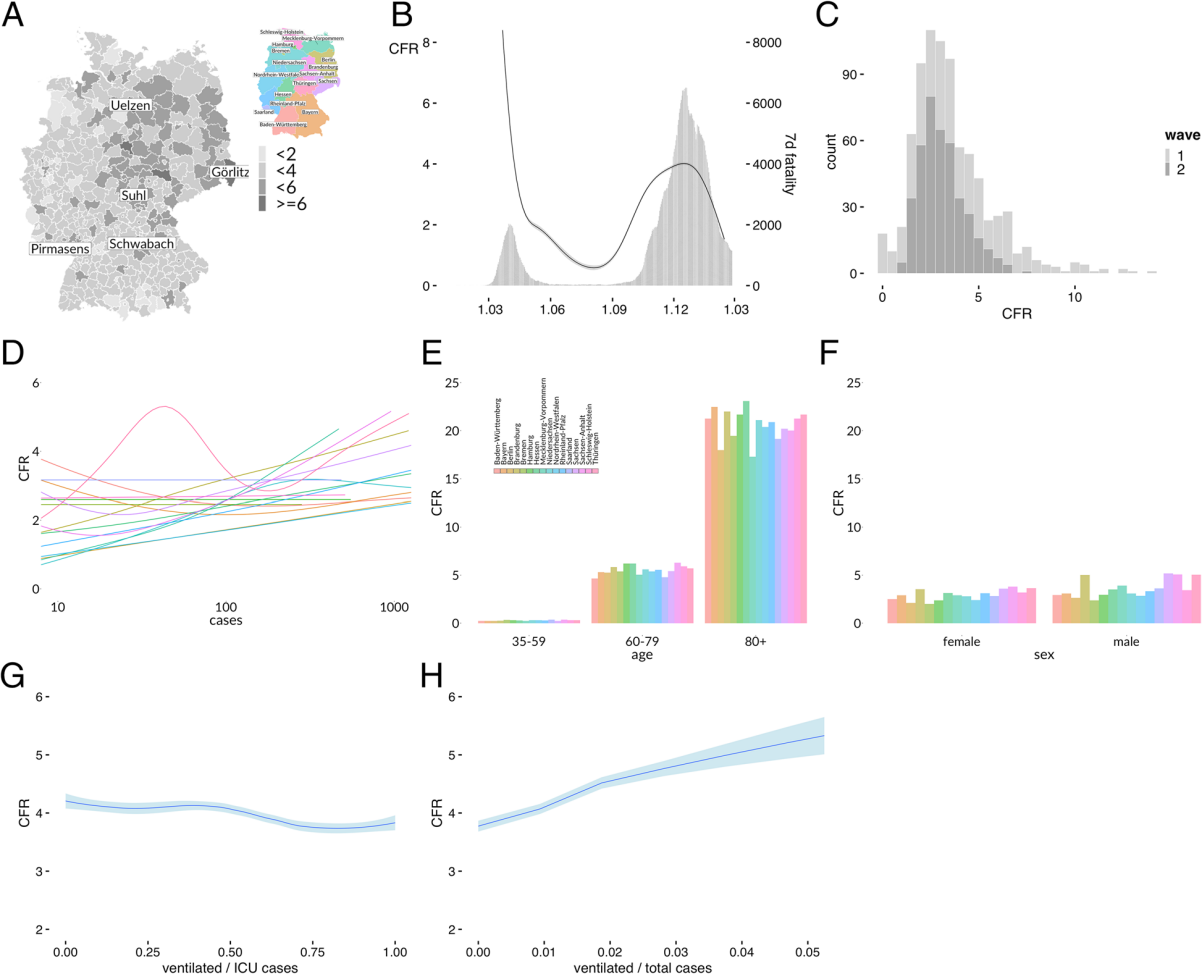
**A** COVID-19 Case Fatality Rate (CFR) map by German districts. Germany is divided into 16 countries (insert) and 402 districts (main figure). The CFR is calculated for the whole period between 2020–01-27 and 2021–02-15. Categories are low (< 2%), average (< 4%) and high > 4%. Data source: Official case and death counts by Robert Koch Institute Berlin. **B** Time course of COVID-19 CFRt in Germany. The CFRt is calculated in a moving window in each German district where all fatalities two weeks before the index day are divided by all new cases during the two preceding weeks. Following irregular high values at the beginning of the pandemic CFRt drops to more realistic values but increases again during the second wave. Weekly case fatalities are shown as vertical bars. For individual country figures see the supplement. **C** Distribution of COVID-19 CFR by German districts. While the peak CFR values are similar during both waves, there is a large tail to the right during the first wave indicating a high heterogeneity. **D** Correlation of COVID-19 CFR and case count by German states. Locally estimated scatterplot smoothing. Colour codes as in A, for individual state figures see the supplement. Most states do not show any correlation with case count, except Lower Saxony, Rhineland-Palatinate and North Rhine-Westphalia, which may indicate some capacity problems to deal with an increasing amount of patients. Thuringia shows an isolated peak for unknown reason. **E**. Distribution of COVID-19 CFR and age group by German states. Colour codes as in A. CFR pattern are similar in all age groups, excluding any preference of the elderly in single states. **F**. Distribution of COVID-19 CFR and sex by German states. Colour codes as in A. CFR pattern is largely identical in all states with the known higher prevalence in men. **G**. COVID-19 CFR and MV ratio at ICU during the second wave in Germany. Locally estimated scatterplot smoothing. There is a slight CFR decrease from 4.0 to 3.6. The high CFR variability therefore depends probably on pre ICU conditions. **H**. COVID-19 CFR and MV ratio to all COVID-19 cases in a district during the second wave. Locally estimated scatterplot smoothing. There is a steady increase in CFR from 3.6 to 5.1 in the indicated range

Germany is divided into 16 federal states (Fig. 1A insert) and 402 districts (Fig. 1A main figure). The CFR is estimated to be 2.92% based on 71.965 fatalities and 2.465.407 cases. Following some irregular high values at the beginning of the pandemic the CFRt dropped to more realistic values but increased again during the second wave (Fig. 1B and supplemental Figure). Although the peak CFRt was identical during both waves, there was a large number of high values during the first wave (Fig. 1C). The highest CFR of all districts was observed in Sachsen / Görlitz including 957 deaths (CFR of 6.4%), followed by Thüringen / Suhl including 85 deaths (CFR of 6.4%) and Bayern /Schwabach including 100 deaths (CFR of 6.3%). There seems to be a considerable underreporting of PCR confirmed infections as described in later antibody studies [19] that might have led to inflated CFR estimates at least at the beginning of the first wave.

Since case counts were much higher during the second wave it may be interesting to look for a possible correlation of CFR and case count (Fig. 1D, for individual state figures see the supplement S1). Most states did not show any correlation, except for perhaps some weak increase in Lower Saxony, Rhineland-Palatinate and North Rhine-Westphalia, which could indicate capacity problems to deal with an increasing amount of patients. It may also be interesting to determine if there is a different age distribution by German states (Fig. 1E). The pattern is largely identical in all age groups, excluding any preferential effect on elderly people. Male sex is always leading to a higher CFR (Fig. 1F).

Figure 1G shows the relationship of CFR and MV patients at ICUs during the second wave in Germany. As there is no association at all, the high CFR variability depends on pre ICU conditions. This is shown in Fig. 1H where CFR steadily increases with the number of MV patients in relation to all COVID-19 infections in a district

**Fig. 2:**
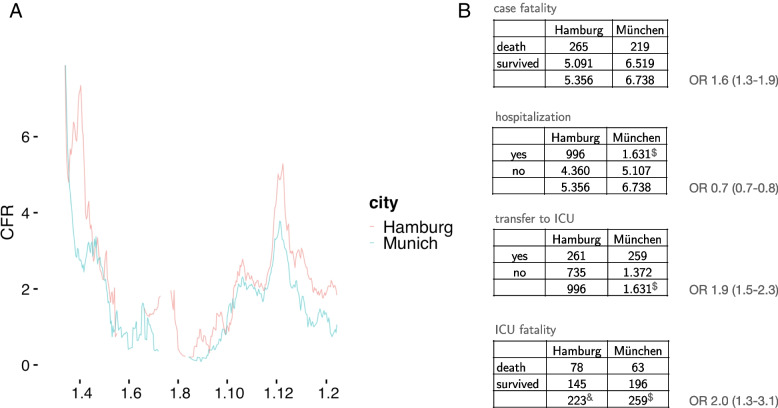
**A** Time course of COVID-19 CFRt in Hamburg and Munich, Germany. The CFRt is calculated as before in a moving window where all fatalities two weeks before the index day are divided by all new cases during the two preceding weeks. Hamburg always showed higher CFRt. Two weeks in July have been excluded from the display as case numbers dropped under 10 per day. **B** COVID-19 case fatalities in the cities Hamburg and Munich during the first wave until 3 June 2020. Data source Roedl et al. 2020 and own data

In a further step the individual time course of the COVID-19 related CFR is compared between major German cities. Berlin is the largest city in Germany but had to be excluded here due to missing data for single districts. Interestingly, Hamburg and Munich, the second and third largest German cities with 1.8 and 1.6 million inhabitants (Fig. 2A) followed different treatment guidelines (Table 1). Although there are more cases in Munich due to the initial outbreak, death risk was found higher in Hamburg (OR 1.6; 1.3–1.9, Fig. 2B). According to official data by the Chamber of Physicians 2019, Hamburg has approximately 50% less physicians working in private praxis which correspond to a lower number of COVID-19 patients sent to hospitals (OR 0.7, 95% CI 0.7–0.8). Hamburg had also less physicians working on general wards which corresponds to a higher number of patients transferred to the ICU (OR 1.9, 95% CI 1.5–2.3). Hence, as also more cases are ventilated at ICUs in Hamburg, this is leading to a higher death rate (OR 2.0, 95% CI 1.3–3.1).

**Table 1:** Treatment Guidelines

**Munich Guideline (author translation)**
Michael Seilmaier, Joachim Meyer, Clemens Wendtner, Niklas Schneider
"Thus, for example we recommend that patients with COVID-19 and respiratory exhaustion in intensive care units first be stabilised by oxygen support via nasal cannula or high flow. The target is an oxygen saturation > 90%. In particular, younger patients under 50 years of age and without severe pre-existing lung disease seem to benefit from the treatment. Invasive ventilations could therefore be avoided, ICU resources could be saved and the length of the stay in the ICU could be significantly shortened."^a^
**Hamburg Guideline (author translation)**
Stefan Kluge, Uwe Janssens, Tobias Welte, Steffen Weber-Carstens, Gereon Schälte, Christoph D. Spinner, Jakob J. Malin, Petra Gastmeier, Florian Langer, Martin Wepler, Michael Westhoff, Michael Pfeifer, Klaus F. Rabe, Florian Hoffmann, Bernd W. Böttiger, Julia Weinmann-Menke, Alexander Kersten, Peter Berlit, Reiner Haase, Gernot Marx, Christian Karagiannidis
"The goal of the therapy for acute hypoxaemic respiratory insufficiency during COVID-19 is to ensure adequate oxygenation. The target should be SpO2 ≥ 90% (COPD patients > 88%) or PaO_2_ > 55 mm Hg. We suggest that for patients with COVID-19 and hypoxaemic respiratory insufficiency (PaO_2_/FiO_2_ = 100–300 mmHg), treatment with high flow oxygen therapy (HFNC) or non-invasive ventilation should be attempted under continuous monitoring and constant intubation standby. We suggest that intubation and invasive ventilation should be considered in patients with COVID-19 and more severe hypoxaemia (PaO_2_/FiO_2_ < 150 mm Hg) and respiratory rates > 30/min. Intubation and invasive ventilation should as a rule be performed when PaO2/FiO_2_ is < 100 mmHg."^b^

^a^ https://www.muenchen-klinik.de/covid-19/knowledge-sharing/

^b^ https://www.awmf.org/leitlinien/detail/ll/113-001.html

**Fig. 3:**
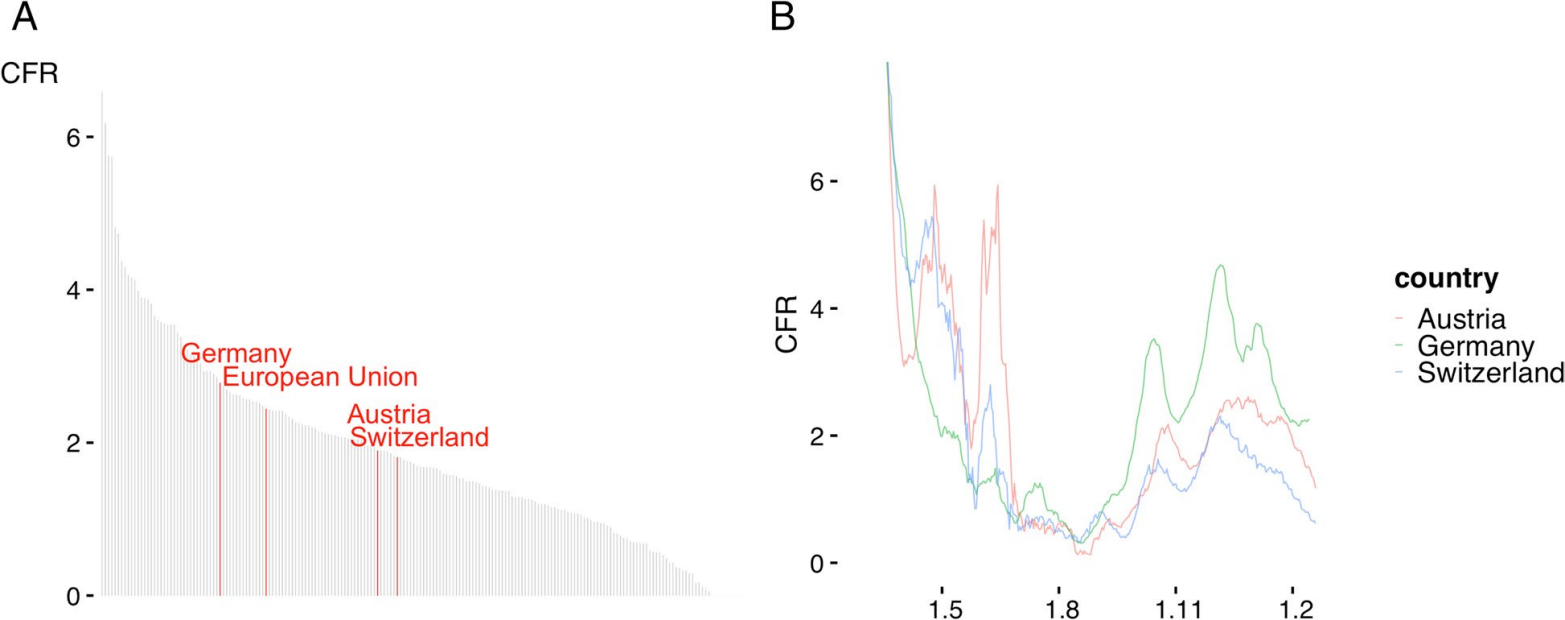
**A** International CFR comparison. Using the OWID database Germany ranks slightly higher than the average EU CFR and considerable higher than Austria and Switzerland. **B** Austria and Switzerland experienced higher CFRt than Germany during the first wave but when changing treatment guidelines, also had lower CFRs during the second wave. Data Source: Official government sources of all three countries

It may be interesting to set these results also in a European context. Germany ranked slightly higher than the EU average and considerably higher than Austria and Switzerland (Fig. 3A and B). Both countries experienced higher CFRs than Germany during the first wave (8 June 2020: Germany 1.3%, Switzerland 2.8%, Austria 4.9%) but after changing treatment guidelines, both countries had lower CFRs during the second wave (2 December 2020: Germany 4.4%, Switzerland and Austria both 2.2%).

## Discussion

COVID-19 related CFR became quickly a central outcome parameter in national and international reviews [20]. More in depth analysis of case fatalities nevertheless remained rare and are even difficult to interpret due to many influencing factors. We now find that the CFR in Germany exceeds not only EU average but also that of neighboring countries that had comparable age structure and health care systems.^1^

Already during the first wave Swiss clinics moved to intermediate care and administered respiratory support including HFNC and NIV) that prevented ICU admission for a large proportion of patients [21]. A rather similar development has been observed in Austria (C. Wenisch, pers comm). Both countries changed initial treatment strategies and, according to a recent press report, cancelled even the order of thousands of respirators that had been placed initially.^2^ This decision was not only based on local experience but also on new studies including a Canadian study that showed an increased hazard of death with each daily increment in driving pressure of mechanical ventilators [22]. Rather similar results were obtained in a Dutch study where the 28-day-mortality significantly increased with tidal volume [23].

In contrast, German CFRs remained high.This has been explained by different diagnostic thresholds whereas we do not find any consistent support for this view (Fig. 1D). Remarkably, the CFR is quite similar also in the younger age group which also argues against a higher affection of nursing homes or different prevalence of DNI/DNR orders. As most people die within hospitals [6] the main reason for the differences is primarily sought by different medical care where early intubation and MV may lead to a worse outcome. Overall ICU fatalities are lower than reported for Germany [6]. The reasons for the discrepancy are not fully clear—there may be some changes over time, there may be more patients dying at home or in nursing homes, there may be more case fatalities that are missed at non university hospitals. We may nevertheless estimate that up to 50% of hospital deaths during the first wave could have been avoided by more conservative treatment.

The exact numbers of ventilated patients have not been released so far. According to figures shown in [24] approximately 65% of ICU patients were ventilated in April during the peak of the first wave and about 55% during the peak of the second wave in December. The ICU outcome remained basically the same with 45% and 40% ICU patients dying in the first and second wave respectively [24]. The improvement in COVID-19 therapy therefore only depends on better initial clinical management as even transfer to ICU dropped from 30 to 14%.

A semi-ecological analysis like the one presented here may of course be influenced by various factors that could not be accounted for. Although consistent, some results may be distorted by improper definitions where standard, intermediate and intensive care are sometimes overlapping. Short-time MV may have different effects than long-time MV. Furthermore there are possible confounding factors; regional outbreaks may inadvertently distort results or there might be different groups of the population being affected at different phases of the pandemic. There are also known issue with data quality and missing values. Although standardized and laboratory confirmed, the exact definition of COVID-19, associated death may vary between states, and there are known differences in SARS-COV2 testing capacity which all may have influenced both numerators and denominators.

Without doubt there are structural differences also between clinical departments where highly specialized centres with the capacity of ECMO therapy may get more seriously ill patients. To exclude that distortion the two second largest cities in Germany have been chosen as comparable units. TUM, where most patients have been treated in Munich, favoured a more conservative management of COVID-19 associated hypoxemia, while Hamburg clinics followed a more aggressive scheme of mechanical ventilation [10]. This may be concluded from numerous external and internal documents (Table 1) as NIV or HFNC is seen more as a bridging therapy in Hamburg clinics. Results obtained here underline the advantage of a less aggressive approach. The Munich approach originates from infectious diseases and pulmonology while Hamburg centers are more dominated by anaesthesiology. Patient centric NIV based therapy is also more demanding needing more personnel and experience to be applied correctly [25]. Although the ultimate goal of maintaining good oxygenation may be identical, fixed blood gas thresholds may be misleading in COVID-19 patients who have been described as "happy hypoxics" [26] as they can tolerate lower pO_2_ values than previously anticipated [27]. In the beginning of the pandemic, patients were also intubated not only in their own interest but also to reduce environmental virus contamination. Evidence is now accumulating that MV may have a negative effect of SARS-COV2 induced Acute Respiratory Distress Syndrome ARDS [28, 29].

Taken together, the results show a good agreement between the clinical and epidemiological findings [30] and provide further evidence that the high CFR in some German hospitals is caused by overtreatment [8, 31]. As randomized clinical trials are largely impossible, also routinely collected data [32] as being provided here, support the clinical observation that less invasive measures like HFNC can reduce the high CFR in ventilated COVID-19 patients. The current more conservative management therefore seems to be justified.

## Supplementary Information

**Additional file 1:** **S1.** Supplement to 1B. Time course of COVID-19 CFRt in Germany broken down by state. **S2.** Supplement to 1D. Correlation of COVID-19 CFR in Germany broken down by state.

## Data Availability

All datasets and analysis code is available from the corresponding author; data and code of FIG 1F and G is immediately available at https://github.com/under-score/Covid-19-ICU

## Footnotes

1 https://www.reuters.com/article/us-health-coronavirus-ventilators-specia-idUSKCN2251PE

2 https://www.nzz.ch/wirtschaft/schweiz-ploetzlich-zu-viele-beatmungsgeraete-ld.1593268
