# Supplementary material for: High variability of COVID-19 case fatality rate in Germany
Source: BMC Public Health. 2023 Mar 2;23:416. doi: 10.1186/s12889-023-15112-0 (PMC9977635; doi:10.1186/s12889-023-15112-0)
Supplement: Supplementary file 1 — Additional file 1: S1. Supplement to 1B. Time course of COVID-19 CFRt in Germany broken down by state. S2. Supplement to 1D. Correlation of COVID-19 CFR in Germany broken down by state. [file 12889_2023_15112_MOESM1_ESM.pdf]

Supplement

S1. Supplement to 1B. Time course of COVID-19 CFRt in Germany broken down by state.

S2. Supplement to 1D. Correlation of COVID-19 CFR in Germany broken down by state.

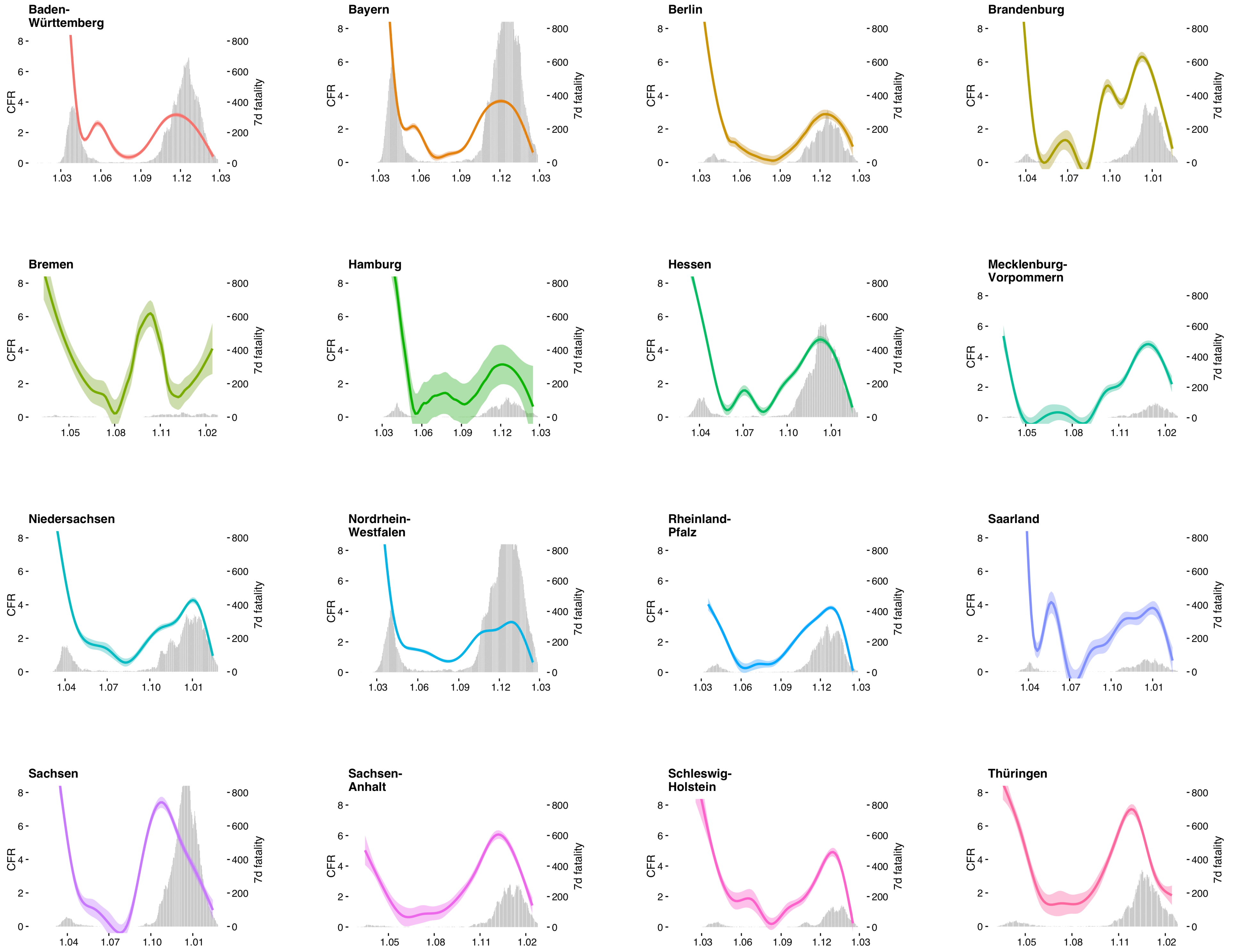
Baden-Württemberg
Bayern
Berlin
Brandenburg
Bremen
Hamburg
Hessen
Mecklenburg-Vorpommern
Niedersachsen
Nordrhein-Westfalen
Rheinland-Pfalz
Saarland
Sachsen
Sachsen-Anhalt
Schleswig-Holstein
Thüringen
CFR
7d fatality
0
2
4
6
8
0
200
400
600
800
1.03
1.06
1.09
1.12
1.03
1.04
1.07
1.10
1.01
1.05
1.08
1.11
1.02

S1

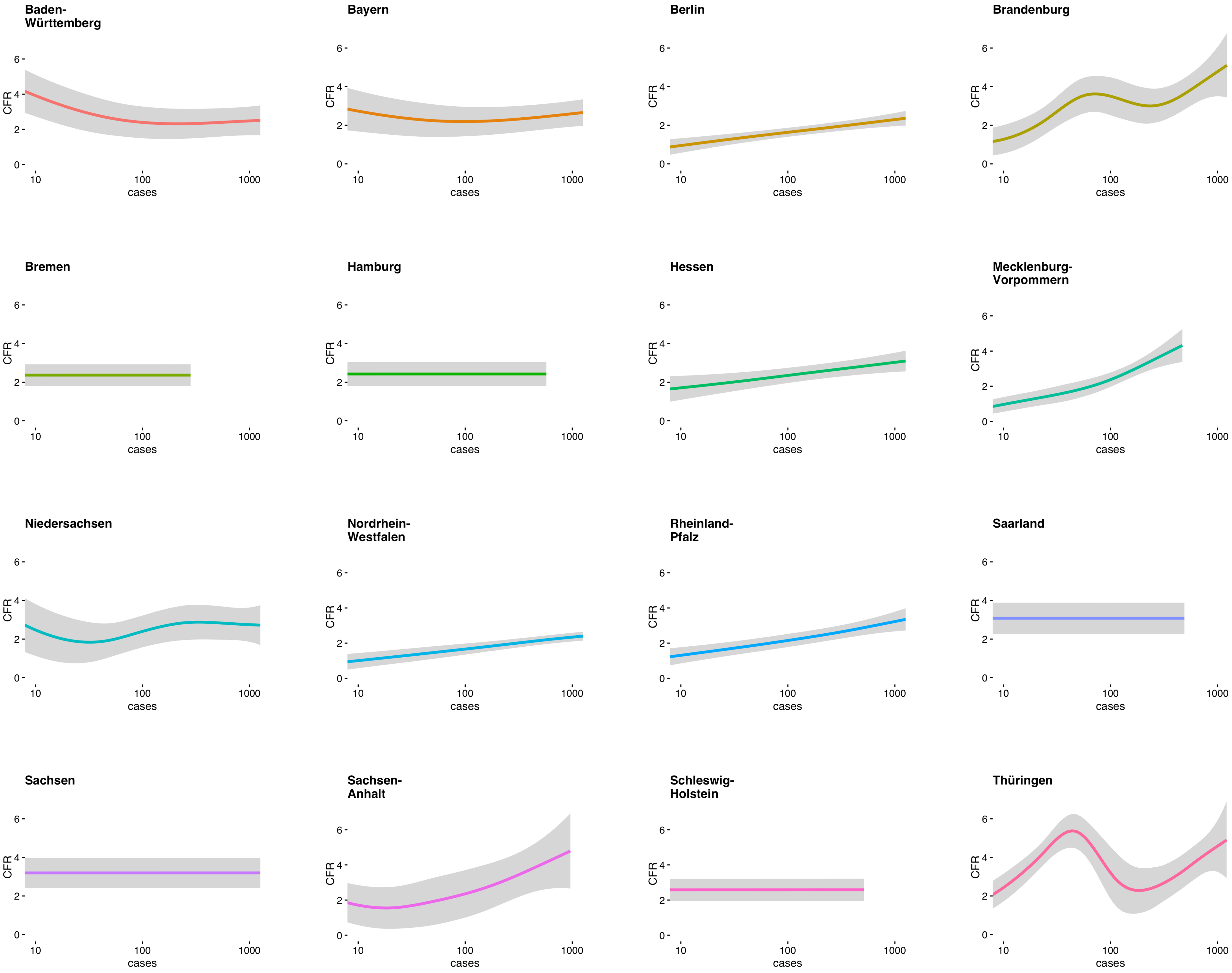
Baden-Württemberg
Bayern
Berlin
Brandenburg
Bremen
Hamburg
Hessen
Mecklenburg-Vorpommern
Niedersachsen
Nordrhein-Westfalen
Rheinland-Pfalz
Saarland
Sachsen
Sachsen-Anhalt
Schleswig-Holstein
Thüringen
CFR
0
2
4
6
cases
10
100
1000

S2
